# Potential of *Camellia sinensis* proanthocyanidins-rich fraction for controlling malaria mosquito populations through disruption of larval development

**DOI:** 10.1186/s13071-016-1789-6

**Published:** 2016-09-22

**Authors:** Jackson M. Muema, Joel L. Bargul, Steven G. Nyanjom, James M. Mutunga, Sospeter N. Njeru

**Affiliations:** 1 Department of Biochemistry, Jomo Kenyatta University of Agriculture and Technology, P.O. Box 62000-00200, Nairobi, Kenya; 2 Molecular Biology and Bioinformatics Unit, International Centre of Insect Physiology and Ecology, P.O. Box 30772-00100, Nairobi, Kenya; 3 Malaria Research Programme, International Centre of Insect Physiology and Ecology, P.O. Box 30772-00100, Nairobi, Kenya; 4 Present Address: Fritz Lipmann Institute (FLI) - Leibniz Institute for Age Research, D-07745 Jena, Germany

**Keywords:** *Camellia sinensis*, Proanthocyanidins, *Anopheles gambiae* (*sensu stricto*), *Anopheles arabiensis*, Larvicidal activity, Vector control

## Abstract

**Background:**

*Anopheles arabiensis* and *A. gambiae* (*sensu stricto*) are the most prolific Afrotropical malaria vectors. Population control efforts of these two vectors have been hampered by extremely diverse larval breeding sites and widespread resistance to currently available insecticides. Control of mosquito larval stages using bioactive compounds of plant origin has the potential to suppress vector populations leading to concomitant reduction in disease transmission rates. In this study, we evaluated the efficacy of *Camellia sinensis* crude leaf extract and its fraction against the larvae of *A. arabiensis* and *A. gambiae* (*s.s*.).

**Methods:**

Late third/early fourth instar larvae (L3/L4) of *A. arabiensis* and *A. gambiae* (*s.s*.) were exposed to increasing doses of *C. sinensis* leaf extract and its active fraction for 72 h, with mortality rates recorded every 24 h in both control and test groups. Ultra performance liquid chromatography electron spray ionization quadruple time of flight coupled with mass spectrometry (UPLC/ESI-Qtof/MS) was used to determine the main active constituents in the fraction.

**Results:**

The major bioactive chemical constituents in the *C. sinensis* leaf extract were identified to be proanthocyanidins. The extract significantly interfered with larval survival and adult emergence in both species (ANOVA, *F*_(5,24)_ = 1435.92, *P <* 0.001). Additionally, larval exposure to crude extract at 250 ppm and 500 ppm for 24 h resulted in larval mortality rates of over 90 % in *A. gambiae* (*s.s*.) and 75 % in *A. arabiensis*. A relatively lower concentration of 100 ppm resulted in moderate mortality rates of < 50 % in both species, but induced growth disruption effects evident as abnormal larval-pupal intermediates and disrupted adult emergence. The estimated LC_50_ concentrations of the crude leaf extract against *A. arabiensis* and *A. gambiae* (*s.s*.) larvae at 24 h were 154.58 ppm (95 % CI: 152.37–158.22) and 117.15 ppm (95 % CI: 112.86–127.04), respectively. The bioactive polar fraction caused 100 % larval mortality in both vector species at 25 ppm.

**Conclusions:**

Our findings demonstrate the potential of green tea extract and its active constituents in disrupting mosquito larval development. This could contribute to the control of mosquito populations and improved management of malaria.

## Background

*Anopheles arabiensis* and *A. gambiae* (*s.s*.) are the principal malaria vectors in Africa accounting for 88 % of the newly-reported global malaria cases and 90 % of the estimated death toll in 2015, impeding socio-economic growth in resource-constrained developing countries [1]. The diversity of mosquito breeding habitats hinders larval control efforts [2]. Currently, malaria vector control relies on integrated vector management (IVM) approaches such as targeting adult stages using long-lasting insecticide treated nets (LLINs) and indoor residual spraying (IRS), environmental management and larval source reduction [1, 3, 4]. Substantial reduction of mosquito populations has been achieved by targeting behavioral attributes of the adult mosquitoes and destruction of larval breeding sites [5]. However, evolution of insecticide resistance in mosquito vectors due to persistent application of chemical insecticides poses a great threat to elimination of malaria from endemic regions [6, 7]. Virtually all the vector control strategies have limitations prompting the need for application of multiple control methods to reduce malaria transmission rates as well as offset insecticide resistance [8, 9]. Thus, there is renewed interest in the search for novel chemicals that have reduced chances of development of resistance, especially ecofriendly natural compounds that are less toxic, or selectively toxic to mosquitoes [10].

Since ancient times, plants have been rich sources of effective natural insecticides [11]. For instance, Neem (*Azadirachtica indica*) derivatives are known to repel as well as inhibit growth in mosquitoes [12, 13]. p-Menthane-3,8-diol (PMD) from *Eucalyptus* plants elicit strong repellent effect against human biting mosquitoes [14]. Proanthocyanidins (condensed tannins) are among the allelochemicals produced by *Camellia sinensis* for defense against herbivory attacks [15]. These are oligomeric or polymeric products of auto-oxidation of flavan-3-ol catechins [16]. Proanthocyanidins are potent antioxidants possessing anti-cancer, anti-inflammatory, antibacterial, antiviral, nematicidal, anti-allergic, cardioprotective and cholesterol reducing activities [17, 18]. Ingestion of proanthocyanidins elicits deleterious effects on herbivory insects by attacking their midgut epithelia following breakdown into free radicals [19].

The immature mosquito stages breed in diverse habitats that may influence their vector competence [20, 21]. Bio-physicochemical parameters of breeding habitats have effects on the larval productivity and vector population dynamics [22]. Perturbation of these habitat parameters with bioactive agents has been reported to significantly suppress mosquito populations [23] and can negatively impact vector competence and life history traits of resultant adult mosquitoes [24, 25]. Thus, targeting the immature stages of mosquitoes can be a viable approach towards elimination of malaria [26, 27].

Several studies have focused on the pharmacological potential of green tea phytochemicals particularly prevention and treatment of cancer, microbial infections, malaria, arthritis, cardiovascular diseases, inflammation, neurodegenerative diseases and other oxidative stress related diseases [28]. Currently, little is known about their potential role in control of medically important insect vectors. Our present study was motivated by previous reports that demonstrated the inhibitory effect of green tea extracts on larval development in *Drosophila melanogaster*, *Aedes aegypti*, *Ae. albopictus* and *Culex quinquefasciatus* [29, 30]. Further, green tea polyphenols have been shown to cause deleterious effects on the development and reproduction fitness of *D. melanogaster* [31]. Therefore, we designed a study to evaluate the efficacy of green tea leaf extract and its constituents against immature larval stages of *A. arabiensis* and *A. gambiae* (*s.s*.). Larvicidal and growth disruption effects of crude green tea leaf extract and its bioactive fraction against the mosquito immature stages were assessed. Our findings show that proanthocyanidins present in green tea extract reduced larval survival and adult emergence in both mosquito species by 100 % at 25 ppm. A sublethal dose of 5 ppm induced growth disruption effects resulting in abnormal larval-pupal intermediates and abortive adult emergence.

## Methods

### Experimental insects

Late third and/or newly emerged fourth instar (L3/L4) larvae of *A. arabiensis* and *A. gambiae* (*s.s*.) were used for experimental studies. These larvae were obtained from a mosquito culture maintained at the International Centre of Insect Physiology and Ecology (*icipe*), Nairobi, Kenya. To establish an experimental larval colony, eggs oviposited on moist filter papers were placed into hatching trays containing 0.5 L dechlorinated water at 28 ± 2 °C. Following egg hatching, the mosquito larvae were kept in plastic trays (39 W × 28 L × 14 D cm) at densities of approximately 300 larvae per litre of distilled water and maintained under the following environmental conditions; 28 ± 2 °C water temperature, 12 L:12D photoperiod and 55–60 % relative humidity. Larvae were fed with 0.3 mg Tetramin® fish meal (Tetra GmbH, Melle, Germany) once every 3 days.

### Collection of plant material and extraction

Fresh immature leaves of green tea, *C. sinensis* (clone TRFK 6/8), were collected from Limuru Archdiocesan Farm (Limuru, Kenya; GPS coordinates: 01°07′10″S, 036°39′37″E; 2,225 m above sea level) in February 2016 with permission to use the plant for this study from Archdiocesan farmers. The tea leaves were shade-dried at 25 ± 2 °C with intermittent aeration to a constant weight. Air-dried tea leaves were milled into powder using an electric grinder (Model 5657; Retsch GmbH, Haan, Germany). Five hundred grams (500 g) of the pulverized leaf powder was infused in 2 L of 90 % methanol for 72 h with intermittent shaking. The extract was filtered with Whatman 1 filter paper (Whatman Inc., Haverhill, USA) and excess solvent removed *via* rotor evaporation. The residual extract was lyophilized in a freeze dryer (Labconco stoppering tray dryer, Labconco Corporation, USA) programmed at 13 °C temperature, vacuum pressure of 998 × 10^-3^ millibars and collector at -40 °C. The resultant extract was stored at -20 °C until use.

### Fractionation of crude green tea extract

Fractionation of active constituents in the crude leaf extract was performed on silica-packed column chromatography. Silica (200 g; Kiesegel 60 M [0.004–0.063 mm mesh size]; Macherey-Nagel GmbH & Co.KG, Düren, Germany) was packed in 40 × 330 mm column and conditioned with analytical grade n-hexane (Sigma Aldrich, St. Louis, USA) for 3 h prior sample loading. Thirty-five grams of the leaf extract were loaded onto the packed silica and elution of various fractions achieved through gradient mobile phase of analytical grade n-hexane and ethyl acetate (100:0–0:100) and finally methanol (Sigma Aldrich, St. Louis, USA). Fractions were chromatographed on thin layer chromatography (TLC) silica plates (ALUGRAM® Xtra SIL G/UV_254_ [0.2 mm], Macherey-Nagel GmbH & Co.KG, Düren, Germany) developed with n-hexane and ethyl acetate (1:2 *v/v*) as mobile phase. The plates were air-dried, sprayed with 30 % sulfuric acid and baked in an oven for detection under UV lamp (λ_254–365 nm_). Based on TLC monitoring and evaluations, fractions with similar retention factor (Rf) values were pooled together, rotor-evaporated and assayed for activity against mosquito larvae.

### Larvicidal activity of green tea extract

Larvicidal assays were conducted following the World Health Organization (WHO) guidelines [32]. Batches of twenty (20) L3/L4 instar mosquitoes were placed in 250 ml glass beakers containing 100 ml of different concentrations of leaf extract (i.e. 500 ppm, 250 ppm, 100 ppm, 50 ppm and 25 ppm) and its fraction (25 ppm, 10 ppm, 5 ppm, 2.5 ppm and 1 ppm). Five replicates were set for each treatment dose (*n* = 100 larvae) and an untreated control (*n* = 100 larvae) was included in each experiment. The doses were formulated by separately dissolving 250 mg, 125 mg, 50 mg, 25 mg and 12.5 mg of leaf extract and 12.5 mg, 5 mg, 2.5 mg, 1.25 mg and 0.5 mg of active fraction in 1 ml of reagent grade absolute ethanol (Fisher Scientific, Loughborough, UK) and diluting into respective doses with 499 ml distilled water. The negative control experiment was set up by placing larvae in 0.2 % of ethanol diluted in distilled water.

### Quantification of larval mortality rates

Equal starting numbers of larvae (*n* = 20 larvae) were placed into each beaker containing different concentrations of leaf extract and active fractions. Mortality rates of treated larvae were quantified at 24 h intervals. Each larva was examined and considered dead if it did not respond to probing with a dropper. Morphological defects of larvae induced by treatment with tea leaf extract relative to controls were analyzed using light microscopy at 25× magnification (Leica Corporation, Heerbrugg, Switzerland). High resolution images of larvae were captured and recorded for further analysis.

### UPLC/ESI-Qtof/MS analysis

In order to re-dissolve the bioactive fraction, 1.5 mg was mixed with 1 ml of LC-MS grade CHROMASOLV methanol (Sigma-Aldrich, St. Louis, USA) and centrifuged at 14000× rpm for 5 min, after which 0.2 μl of the supernatant was automatically injected into UPLC/ESI-Qtof/MS. The chromatographic separation was achieved on a Waters ACQUITY UPLC (ultra-performance liquid chromatography) I-class system (Waters Corporation, Milford, USA) fitted with a 2.1 mm × 50 mm, 1.7 μm particle size Waters ACQUITY UPLC BEH C18 column (Waters Corporation, Dublin, Ireland) heated to 40 °C and autosampler tray cooled to 5 °C. Mobile phases of water (A) and acetonitrile (B) each containing 0.01 % formic acid were employed. The following gradient was used: 0–5 min, 10 % B; 5–7 min, 10–60 % B; 7–10 min, 60–80 % B; 10–15 min, 80 % B; 15–18 min, 100 % B; 18–20 min, 100 % B; 20–21.5 min 100–10 % B; 21.5–25 min 10 % B. The flow rate was held constant at 0.4 ml min^-1^. The UPLC system was interfaced with electrospray ionization (ESI) to a Waters Xevo Qtof-MS operated in full scan MS^E^ in positive mode. Data were acquired in resolution mode over the *m/z* range 100–1,200 with a scan time of 1 s using a capillary voltage of 0.5 kV, sampling cone voltage of 40 V, source temperature 100 °C and desolvation temperature of 350 °C. The nitrogen desolvation flow rate was 500 l/h. For the high-energy scan function, a collision energy ramp of 25–45 eV was applied in the T-wave collision cell using ultrahigh purity argon (≥99.999 %) as the collision gas. A continuous lock spray reference compound (leucine enkephalin; [M + H]^+^ = 556.2766) was sampled at 10 s intervals for centroid data mass correction. The mass spectrometer was calibrated across the 50–1,200 Da mass range using a 0.5 mM sodium formate solution prepared in 90:10 2-propanol/water (*v/v*). Mass Lynx version 4.1 SCN 712 (Waters Corporation, Milford, USA) was used for data acquisition and processing. The elemental composition was generated for every analyte. Potential assignments were calculated using mono-isotopic masses with a tolerance of 10 ppm deviation and both odd- and even-electron states possible. The number and types of expected atoms was set as follows: carbon ≤ 100; hydrogen ≤ 100; oxygen ≤ 50; nitrogen ≤ 6; sulfur ≤ 6. The UPLC/ESI-Qtof/MS data acquisition and analysis were based on the following parameters: mass accuracy (ppm) = 1,000,000 × (calculated mass-accurate mass)/calculated mass; fit conf % is the confidence with which the measured mass (accurate mass) matches the theoretical isotope models of the elemental composition in the list; elemental composition is a suggested formula for the specified mass. This reflects a summation of the quantities of elements, isotopes or superatoms that can comprise the measured data, calculated using the following atomic masses of the most abundant isotope of the elements: C = 12.0000000, H = 1.0078250, *N* = 14.0030740, O = 15.9949146, S = 31.9720718. The empirical formulae generated were used to predict structures and proposed based on the online databases (Chemspider, Metlin) and published literature [33, 34].

### Data analysis

Data were entered and organized in Microsoft Excel 2010 spreadsheet then exported to R software version 3.2.3 (The R Project for Statistical Computing, www.r-project.org) for analyses. Corrected mortality rates were expressed as % mean ± standard deviation (SD) for each tested dose. The test concentrations were log_10_-transformed to reduce variation prior to fitting a dose-response model for estimating lethal dose concentrations. Non-linear regression using *glm* function in R with *probit* link and quasi binomial distribution error was used to estimate the lethal concentrations of crude extract and its active fraction. LC_50_ of both the crude extract and its active fraction were estimated from the *glm* output using the *dose.p* function in MASS Package in R. The significance of differences between treatment means was determined by analysis of variance (ANOVA) with values of *P* < 0.05 considered statistically significant. Graphs were designed using GraphPad Prism version 7.0 for Windows (GraphPad Software, San Diego, USA). Images were processed using publicly-available IMAGEJ software (National Institutes of Health, https://imagej.nih.gov/ij/).

## Results

### Phytochemistry

**Fig. 1:**
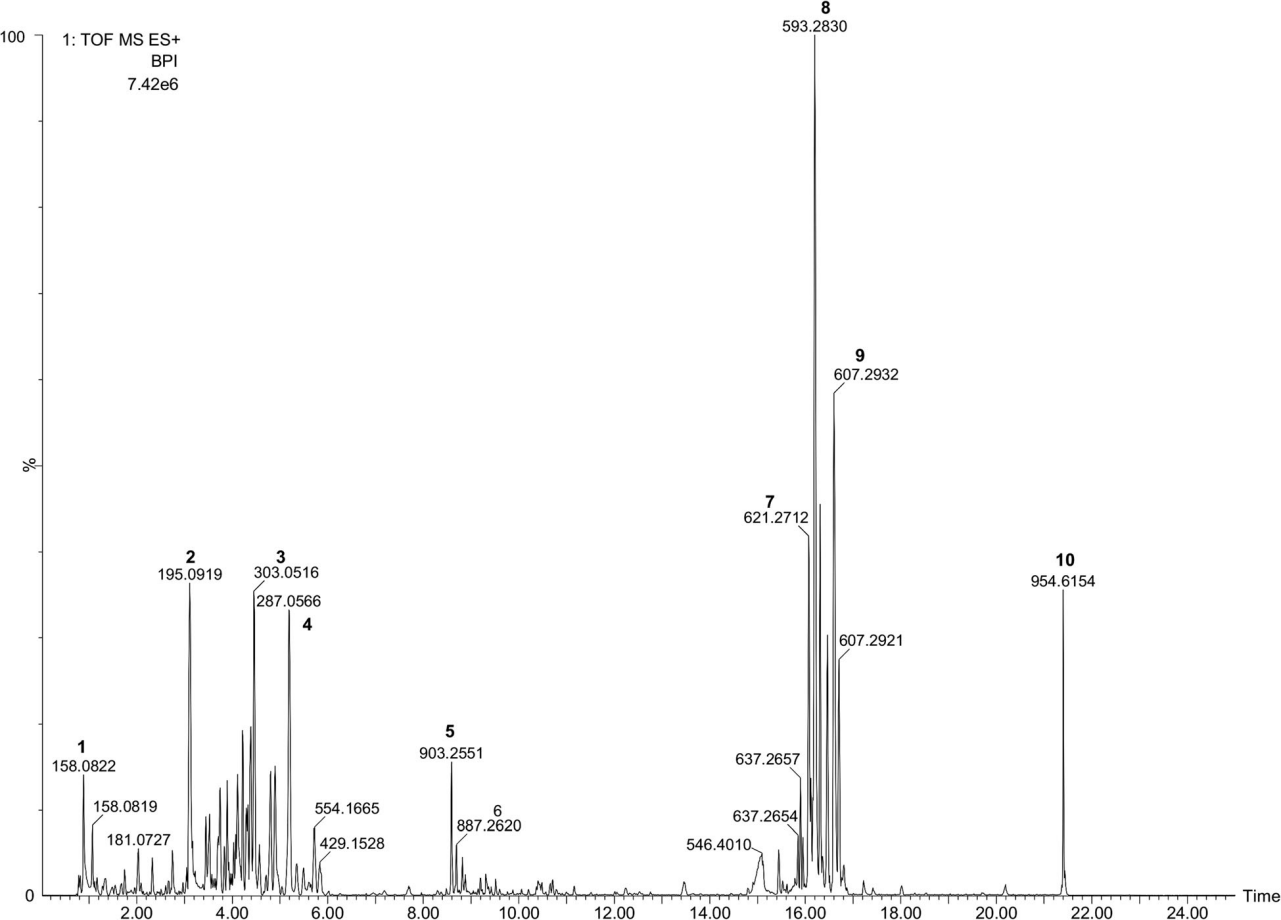
UPLC/ESI-Qtof/MS analysis profile of bioactive fraction of *Camellia sinensis* leaf extract. Six bioactive compounds were detected as follows: **2**-Caffeine, **3**-Quercetin, **4**-Kaempferol, **5**-Kaempferol rhamnoside, **6**-Kaempferol rhamnosyl glucoside, **8**-Proanthocyanidin. Proanthocyanidin m/z 593.2830 was the most abundant compound (15.2641 %)

Phytochemical analysis of the bioactive methanolic fraction detected 6 major biologically-active compounds, in addition to a protein and 3 unknown compounds (Table 1). Proanthocyanidins (C_31_H_28_O_12_) *m/z* [M/H]^+^ 593.2830 (15.2641 %) were detected through UPLC/ESI-Qtof/MS as the most abundant compounds in the bioactive fraction of tea extract (Fig. 1). Other prominent mass spectrum peaks that denote bioactive compounds were *m/z* 195.0919 (C_8_H_11_N_4_O_2_) (**2**), 303.0516 (C_15_H_10_O_7_) (**3**), 287.0566 (C_15_H_10_O_6_) (**4**), 903.2551 (C_42_H_46_O_22_) (**5**) and 887.2620 (C_42_H_46_O_21_) (**6**). A database search tentatively identified the compounds represented by peak **2** (R_t_ 3.06 min) as Caffeine, peak **3** (R_t_ 4.42 min) Quercetin, peak **4** (R_t_ 5.10 min) Kaempferol, peak **5** (R_t_ 8.56 min) Kaempferol rhamnoside and peak **6** (R_t_ 8.56 min) Kaempferol rhamnosyl glucoside (Table 1 and Fig. 1).

**Table 1:** Tentative identification of the constituents of *C. sinensis* bioactive fraction. Data show tentative identification of compounds within the bioactive fraction of *C. sinensis* extract from published literature and publicly accessible online databases, monoisotopic mass m/z, chemical formula, and peak area of each compound at a particular retention time (R_t_)

No.	R_t_ (min)	*m*/*z* [M + H]^+^	Peak area (%)	Chemical formula	Tentative identification
1	0.84	158.0822	2.1269	–^a^	Unknown^b^
2	3.06	195.0919	7.0454	C_8_H_11_N_4_O_2_	Caffeine
3	4.42	303.0516	4.9190	C_15_H_10_O_7_	Quercetin
4	5.10	287.0566	6.0235	C_15_H_10_O_6_	Kaempferol
5	8.52	903.2551	1.4543	C_42_H_46_O_22_	Kaempferol rhamnoside
6	8.56	887.2620	0.6415	C_42_H_46_O_21_	Kaempferol rhamnosyl glucoside
7	16.00	621.2712	6.3769	^−^	Unknown
8	16.15	593.2830	15.2641	C_31_H_28_O_12_	Proanthocyanidin
9	16.52	607.2932	9.4702	C_36_H_38_N_4_O_5_	Phenyl peptide
10	21.44	954.6154	2.3624	–	Unknown

^a^–Represents missing chemical formula for the compounds with serial number 1, 7 and 10

^b^‘Unknown’ means that compound was unidentifiable from searched databases and published literature

### Toxicity effects of leaf extract and its fraction on mosquito larvae

Larval survival and adult emergence was significantly reduced when L3/L4 instars of *A. arabiensis* and *A. gambiae* (*s.s*.) were exposed to different concentrations of crude extract and its bioactive fraction (ANOVA, *F*_(5,24)_ = 1435.92, *P <* 0.001). The untreated control group achieved 100 % survival rates for the entire analysis period. Higher extract doses (250 ppm and 500 ppm) evoked more than 90 % larval mortality in *A. gambiae* (*s.s*.) and 75 % in *A. arabiensis* at 24 h post-exposure (Table 2). At 24 h post-exposure, the estimated LC_50_ dose and its associated 95 % confidence intervals for *A. arabiensis* was 154.58 ppm (152.37–158.22), and for *A. gambiae* (*s.s*.) was 117.15 ppm (112.86–127.04). Our results indicate that, larval survival rates significantly reduced with prolonged exposure (72 h) relative to controls (ANOVA, *F*_(5,24)_ = 117.64, *P <* 0.001; Table 2). A sublethal dose of extract at 100 ppm induced growth disruption effects characterized by: failure of larvae to molt into pupae, resulting into abnormal larval-pupal intermediates (Fig. 3c, d) and death of adults during eclosion from the pupal stage, with their legs and wings stuck in pupal caste (Fig. 3e, f).

**Table 2:** Acute toxicity of crude green tea (*Camellia sinensis*) extract on exposure to L3/L4 instars of *A. arabiensis* and *A. gambiae* (*s.s*.) for 24, 48 and 72 h post-exposure. Summary of percentage mean (± SD) mortality rates of five replicates of mosquito larvae exposed to different concentrations of *C. sinensis* crude leaf extract for different time periods (24, 48 and 72 h). Half maximal lethal concentrations (LC_50_) for each dose exposure period have been determined at their 95 % confidence intervals. The mosquito larvae exhibited significant susceptibility to the bioactive fraction at *P* < 0.05

	Concentration (ppm)						Lethal concentration (ppm)	
Time	500	250	100	50	25	Control	LC_50_	95 % CI
An. arabiensis*								
24 h	86 ± 9.62	75 ± 24.75	30 ± 19.69	0 ± 0.00	0 ± 0.00	0 ± 0.00	154.58	152.37–158.22
48 h	98 ± 4.47	92 ± 5.70	53 ± 20.19	0 ± 0.00	0 ± 0.00	0 ± 0.00	154.58	152.37–158.22
72 h	100 ± 0.00	95 ± 5.00	60 ± 18.03	0 ± 0.00	0 ± 0.00	0 ± 0.00	154.58	152.37–158.22
*An. gambiae* (*s.s*.)*								
24 h	100 ± 0.00	91 ± 9.62	39 ± 6.52	0 ± 0.00	0 ± 0.00	0 ± 0.00	117.15	112.86–127.04
48 h	100 ± 0.00	98 ± 2.24	62 ± 10.37	0 ± 0.00	0 ± 0.00	0 ± 0.00	87.11	82.57–112.82
72 h	100 ± 0.00	100 ± 0.00	84 ± 11.94	0 ± 0.00	0 ± 0.00	0 ± 0.00	87.11	82.57–112.82

Data presented as mean ± SD of five replicates

*Abbreviations*: *LC*_*50*_ lethal concentration that killed 50 % of test mosquito larvae population, *CI* confidence interval

*Mean values are not significantly different *P* ≤ 0.05 (ANOVA)

**Fig. 2:**
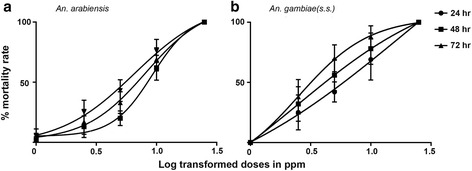
Dose-response curves showing treatment effects of bioactive fraction of *C. sinensis* on *A. arabiensis* and *A. gambiae* (*s.s*.) larvae at 24, 48 and 72 h post-exposure. Doses of the extract are log-transformed. The curves show dose-response fitted models of *A. arabiensis* (**a**) and *A. gambiae* (*s.s*.) (**b**) larvae, treated with bioactive fraction of *C. sinensis* at different exposure time periods (24, 48 and 72 h). Each point on the curve represents percentage mean (± standard deviation, SD) larval mortality of five replicates for a particular dose

**Fig. 3:**
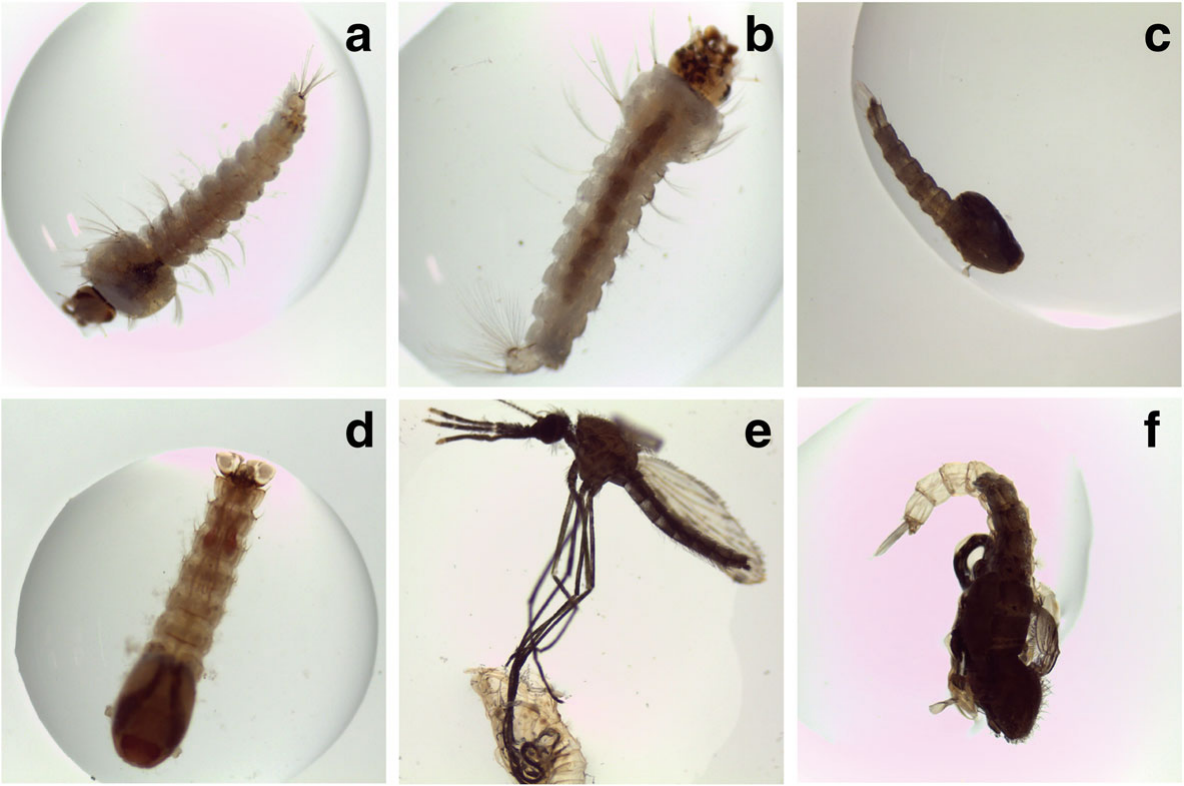
Growth disruption effects mediated by *Camellia sinensis* extract at 72 h post-exposure. **a** *Anopheles gambiae* (*s.s*.) control larva. **b** *Anopheles arabiensis* control larva. **c** Abnormal *A. gambiae* (*s.s*.) larval-pupal intermediate. **d** Abnormal *An. arabiensis* larval-pupal intermediate. **e** Aborted adult emergence in *A. gambiae* (*s.s*.) with legs stuck in pupal caste. **f** *An. arabiensis* adult emergence arrested (visualization of the changes in larval morphology before and after treatment with extract was monitored using light microscopy at 25× magnification)

Further, we investigated the larvicidal efficacy of the bioactive fraction by exposing the larvae to 25 ppm, 10 ppm, 5 ppm, 2.5 ppm and 1 ppm. Maximum larval mortality rates of 100 % were attained at 25 ppm treatment dose within 24 h incubation in both *A. arabiensis* and *A. gambiae* (*s.s*.) (Table 3 and Fig. 2). Moderate larval toxicity that increased with incubation duration reaching peak levels at 72 h post-exposure occurred at 2.5, 5 and 10 ppm, whereas minimal toxicity was recorded at 1 ppm exposure. Growth disruption effects similar to those induced by the crude extract at 100 ppm were observed at 5 ppm (Fig. 3). Differences between the treatment means were not statistically significant (ANOVA, *F*_(5, 24)_ = 85.33, *P* < 0.001).

**Table 3:** Acute toxicity in L3/L4 instars of *A. arabiensis* and *A. gambiae* (*s.s*.) resulting from treatment of larvae with the active green tea fraction for 24, 48 and 72 h. Data presented below indicates the percentage means (± S.D) of mortality rates of mosquito larvae exposed to different concentrations of bioactive fraction of *C. sinensis* extract for different time periods (24, 48 and 72 h). Five replicates were included in the study. Half maximal lethal concentrations (LC_50_) for each dose exposure period have been determined at their 95 % confidence intervals. The mosquito larvae exhibited significant susceptibility to the bioactive fraction at *P* < 0.05

	Concentration (ppm)						Lethal concentration (ppm)	
Time	25	10	5	2.5	1	Control	LC_50_	95 % CI
An. arabiensis*								
24 h	100 ± 0.00	62 ± 10.37	20 ± 6.12	13 ± 9.08	0 ± 0.00	0 ± 0.00	7.37	3.98–12.64
48 h	100 ± 0.00	69 ± 9.62	33 ± 9.08	17 ± 10.37	0 ± 0.00	0 ± 0.00	6.22	3.04–11.06
72 h	100 ± 0.00	76 ± 9.62	43 ± 9.08	25 ± 10.00	0 ± 0.00	0 ± 0.00	5.20	2.17–9.70
*An. gambiae* (*s.s*.)*								
24 h	100 ± 0.00	69 ± 17.10	42 ± 8.37	25 ± 14.58	0 ± 0.00	0 ± 0.00	5.52	2.68–9.65
48 h	100 ± 0.00	78 ± 2.24	56 ± 12.94	32 ± 14.40	0 ± 0.00	0 ± 0.00	4.45	1.55–8.71
72 h	100 ± 0.00	88 ± 9.08	70 ± 11.18	38 ± 14.40	0 ± 0.00	0 ± 0.00	3.60	0.29–8.71

Data presented as mean ± SD of five replicates

*Abbreviations*: *LC*_*50*_ lethal concentration that killed 50 % of test mosquito larvae population, *CI* confidence interval

*Mean values are not significantly different *P* ≤ 0.05 (ANOVA)

Treatment of mosquito larvae with sublethal doses of green tea bioactive fraction exerted growth disruption effects after 72 h of exposure (Fig. 3). Progression of metamorphosis, from larva to pupa and finally adult, was halted resulting into abnormal larval-pupal intermediates and arrested adult emergence. Further, some adults that successfully emerged from the larvicide treated water were unable to fly from the test beakers and died on the water surface, a phenomenon similarly observed with crude extract at 100 ppm.

## Discussion

Malaria-transmitting mosquitoes have developed physiological and behavioral mechanisms to successfully evade population control strategies targeting adult stages [7]. Limitations imposed by these vector control tools have necessitated complementary strategies. Larviciding, a less-practiced component of integrated vector management, has traceable history of success in eradication of malaria from Brazil and Egypt and currently revived in some African countries [26, 35]. Mosquito metamorphosis represents a low cost and feasible target of population regulation through interfering with development of immature stages [36]. Despite the dramatic reduction in mosquito populations on persistent application of chemical insecticides, negative implications have limited their continued use [37]. Thus, development of safe, novel and selectively toxic larvicides has been welcomed into integrated mosquito control programmes [38, 39]. Principally, their anticipated multimodal actions against target arthropods limit chances of resistance development and could be a pathway to designing resistant-resilient insecticides [40]. Prospection for use of plant-derived insect growth regulators as biolarvicides has gained considerable attention aimed at disrupting successful development of agricultural and medical nuisance insects including mosquitoes [10, 13, 41].

In this study, we aimed to evaluate whether green tea could be a potential source of mosquito control agents, by investigating its efficacy against larvae of *A. gambiae* (*s.s*.) and *A. arabiensis*. We found that, the initial larval bioassays with crude leaf extract significantly reduced survival and development of larval stages in both mosquito species suggesting presence of biologically active phytochemicals. This necessitated fractionation in order to isolate specific bioactive ingredients, tentatively identified as caffeine, quercetin, kaempferol, kaempferol rhamnoside, kaempferol rhamnosyl glucoside and proanthocyanidins.

Consistent with previous results from studies conducted on other insects [29–31], *C. sinensis* extract exhibited larvicidal activity in addition to induction of growth disruption effects. However, in contrast to our dosage data, these studies reported relatively higher extract dosages of between 10 mg/ml to 75 mg/ml (translating to 10,000–75,000 ppm) to achieve similar larvicidal potency. The varied observations could be attributed to susceptibility difference of test insects, variation in abundance of bioactive constituents in plant extracts, extraction method and geographical location of the studied plants. Contrary to these studies which attributed toxicity effects to polyphenolic constituents of *C. sinensis* especially the abundant (-)-epigallocatechin-3-gallate (EGCG) [31], we found proanthocyanidins to be the abundant bioactive compounds most strongly associated with the observed effects. Surprisingly, catechins were not detected in the bioactive fraction. However, proanthocyanidins are polyphenolic products of catechins epimerization associated with plant defenses [16, 19]. The other compounds identified within the fraction might have contributed towards exerting synergistic or racemic mixture effects to proanthocyanidins.

Caffeine has been reported to interfere with mosquito larval development [42]. The hydroxylflavones, quercetin and kaempferol, induce cell cycle arrest by inhibiting CDC25A tyrosine phosphate at G2/M phase and/or inducing apoptosis [43]. In nature, polyphenolic compounds including proanthocyanidins (condensed tannins) and other flavonoids form part of defense against fungal attacks and insect herbivory in plants [44]. They provoke feeding deterrence with intense disorganization of midgut epithelia cells upon ingestion, which concomitantly reduces insect survival and development [45]. The fact that green tea polyphenolic compounds exert anti-carcinogenic effect by inducing cell cycle arrest, apoptosis and growth inhibition could also be implicated in this study [46, 47]. The post-embryonic stages of insects comprise of cell proliferation and DNA replication events preceding growth and morphogenetic organization [48]. As proanthocyanidins are pro-oxidants and pro-apoptotic molecules that astringently precipitate cellular proteins [49], they could presumably halt these events in developing insects resulting to death. Also, the compounds bind to nucleic acids, increasing topoisomerase II DNA cleavage activity, inducing DNA breaks and reduced cell survival [50, 51]. Taken together, these mechanisms could be attributable to the impaired larval development and toxicity.

Although molecular studies were not included in this study to determine the linkage between the genotype and the observed morphological traits, peculiar observations were noted in regard to developmental phenotypes. Incubation of the mosquito larvae with sublethal dose of 5 ppm of the bioactive fraction induced developmental defects (Fig. 3) similar to those exhibited by insect growth regulators (IGRs) [52, 53]. The transition of mosquito larvae into adult stage was found to fail at larval-pupal intermediates, while others remained permanently stuck as they eclosed. Insect growth regulatory (IGR) compounds act by adversely interfering with physiologically and hormonally-regulated developmental events resulting into immature deaths and non-viable reproductive adults [52, 54]. Commonly known IGRs are insect developmental hormone agonists that mimic the actions of juvenile hormone and ecdysone while others inhibit chitin synthesis [55, 56]. Interestingly, among the compounds tentatively identified in the bioactive fraction, none seemed structurally similar to insect developmental hormones, ecdysteroid 20-hydroxyecdysone (20-E) and sesquiterpenoid juvenile hormone (JH). However, the presence of flavonoid-like polyphenols in the larval breeding water suggested modulated neuroendrocrine signaling networks interfering with larval development [57]. Of importance is insulin/insulin-like pathway, a conserved regulatory signaling pathway that coordinate insect growth and metamorphosis by regulating biosynthesis of development hormones [58–60]. Other studies have documented that phytochemicals especially flavonoid-like polyphenols negatively impact insect molting by interfering with prothoracicotropic hormone and ecdysteroid action resulting to delayed maturity and abnormal developmental phenotypes or mortality [61]. Growth and development transitions in immature insects are orchestrated by morphological and ultra-structural changes regulated by coordinated actions of JH, ecdysone and eclosion hormones [62]. Insulin/insulin-like signaling interplay between the developmental events to ensure static allometry in holometabolous insects [63–65]. Hence, any exogenous agent that interferes with either the signaling networks or homeostasis of the insect developmental hormones might result in abnormal growth and development as observed in Fig. 3.

Plant-based IGRs with similar effects on developing mosquitoes have been previously reported to effectively suppress mosquito populations [66–71]. *C. sinensis* is favoured over other plants as a potential source of mosquito control agents because it is (i) cultivatable (ii) non-endangered plant species, and (iii) its waste can be recycled. Our findings suggest that green tea leaf proanthocyanidins could be employed to control malaria vector populations. It is paramount to conduct studies to determine toxicity effects of proanthocyanidins against non-target organisms prior to commercialization and large scale field application of this product as larvicidal agent. Further, studies encompassing molecular target identification, simulation and ligand docking are recommended to create avenues for synthesizing structurally similar candidate analogs to ease application.

## Conclusion

In Kenya, *C. sinensis* is grown as a major cash crop. We found that the leaf extract and its active constituents have great potential for controlling mosquito larvae. Proanthocyanidins were the abundant compounds tightly associated with toxicity of larvae though the other compounds identified in the extract may contribute to bioactivity. Besides causing larval toxicity, the sublethal doses induced growth disruption effects that inhibited adult emergence.

## Abbreviations

LLINs: Long-lasting insecticide treated nets
IRS: Indoor residual spraying
IVM: Integrated vector management
PMD: p-Menthane-3,8-diol
WHO: World Health Organization
IGRs: Insect growth regulators
